# Bibliometric Analysis of Marine Traditional Chinese Medicine in Pharmacopoeia of the People's Republic of China: Development, Differences, and Trends Directions

**DOI:** 10.1155/2022/3971967

**Published:** 2022-12-27

**Authors:** Wenxuan Cao, Jia Liu, Yulin Dai, Yashuang Zhou, Ruili Li, Peng Yu

**Affiliations:** ^1^Department of Pharmaceutical Science, Changchun University of Chinese Medicine, Changchun 130117, China; ^2^Jilin Ginseng Academy, Changchun University of Chinese Medicine, Changchun, Jilin 130117, China; ^3^Graduate School, Changchun University of Chinese Medicine, Changchun 130117, China

## Abstract

**Background:**

Marine traditional Chinese medicine (MTCM) is a class of traditional medicine that has antitumor, anti-inflammatory, and antiviral properties. Bibliometric approaches were used in this study to conduct systematic research in order to gain a complete picture of MTCM research around the world.

**Methods:**

CiteSpace and NoteExpress software were utilized as tools to examine the information about authors, sources, keywords, etc. Chinese publications were collected from the CNKI, VIP, and WANFANG databases; English publications were collected from the Web of Science database.

**Results:**

A total of 10080 publications were screened, and the search volume of Chinese literature is greater than that of English literature; Nanjing University of Chinese Medicine, China, and Jeju National University, South Korea, published a greater number of articles than other institutions; the scholars Zhaohui-Zhang and Youjin-Jeon have published the highest number of articles in the world. MTCM of shells was often researched for inorganic elements, and data mining methods were applied frequently; MTCM of animals was commonly used for antifatigue and was taken authenticity identification owing to the scarcity of resources; scholars conducted the most research on MTCM of plants, this category usually for antitumor, anti-inflammatory, and antioxidant purposes, and the mechanisms of action were studied in depth. The Chinese literature has undertaken a multifaceted research study based on the theories of processing and the nature of TCM. In the English literature, in-depth studies have been done from the perspectives of the mechanism of action, the extraction and purification of active substances, etc.

**Conclusions:**

According to the analysis of keywords, different medicinal parts present their own special research directions, and different research hotspots have also emerged under different medical theories. The development of MTCM is moving in the direction of standardization and modernization, thanks to the development of cross-disciplinary research as well as the use of several new technologies and statistical techniques.

## 1. Introduction

The ocean accounts for more than 70% of the earth's surface area, and it is the cradle of life and a crucial source of supplies for human nutrition. Due to the particular marine environment of high salt, high pressure, hypoxia, and lack of sunlight, marine organisms and plants often produce a range of secondary metabolites with specific activities during growth and metabolism [1–3]. For example, polysaccharides [4] or macrolides [5] from the ocean have, without a doubt, incalculable value in the medical area. According to the Pharmacopoeia of the People's Republic of China (ChP), marine medicines can be divided into marine traditional Chinese medicines (MTCM), marine chemical medicines, and marine biological products [6]. Among them, MTCM refers to marine natural medicines used for disease prevention and health care under the guidance of traditional Chinese medicine (TCM) theory [7, 8], which include marine botanicals, marine animal medicines, and marine mineral medicines [9]. MTCM, as an important part of traditional Chinese medicine resources, is rich in resources and unique in its therapeutic benefits; its research and development have focused widespread attention on the medical profession [10–12]. China, as the originator of TCM, has been using MTCM to treat aliments for nearly 2,000 years, and many MTCMs have been confirmed to be medically helpful through long-term clinical trials [13]. In 2012, the “Twelfth Five-year Plan” launched in China clearly put forward the requirement of “scientific planning for the development of marine economy, rational exploitation of marine resources, and focus on cultivation and growth of marine biomedicine.” Since the implementation of this plan, MTCM has become a popular research topic in the field of TCM. However, according to the National Oceanic and Atmospheric Administration's estimate that just 20% of the ocean has been explored by humans [14], there is still a large blank space in the human exploration of MTCM.

MTCM is rich in bioactive substances, such as terpenoids, peptides, polyethers, amino acids, lipids, fatty acids, alkaloids, saponins, organic acids, and proteins. For example, algae contain many carbohydrates and polysaccharides [15], and mineral medicine contains many inorganic salts and calcium carbonate components [16]. There are many organohalides and guanidine derivatives in MTCM; however, few are found in terrestrial organisms [17, 18]. For instance, the tetracyclic tetraterpenoids with a novel framework were isolated from *Sarcophyton tortuosum Tixier-Durivault*, which is a chemical found only in the ocean [19]. The pharmacological activity of MTCM is also broad. We had observed in our previous study that the active fucoidan (JHCF4), which is extracted from the crude fucoidan in acid-processed*Hizikia fusiforme*, has antitumor, antioxidant, and maybe hepatoprotective properties against ethanol-induced damage [20–22]. Beyond that, O*streae concha* polysaccharides can boost the immune system [23]; *Sargassum* has effects on antitumor and antiviral therapy [24, 25]; *Hippocampus* has a great effect on antiaging [26]; and in vitro cellular studies have also revealed that water-soluble*Margaritifera concha* protein has a great impact on stimulating the differentiation of bone marrow stromal cells into osteoblasts and increasing osteoblast proliferation [27].

Bibliometric analyses were widely used to gauge the scholarly impact of scientific publications. CiteSpace is JAVA-based virtual environment software developed with the support of Chaomei-Chen, a professor at Drexel University [28]. It was used to find and display new trends and developments in the scientific literature and was also significantly useful software for visual analysis [29, 30]. NoteExpress is a professional-grade literature search tool and management software with core functions of gathering, management, application, and mining of knowledge among other things [31]. Although more and more scholars are engaged in the study of MTCM, bibliometrics has received little attention. We chose the Chinese and English literature as the scope of the study for the first time and applied bibliometric methodologies to conduct a systematical review of the current status and development trend of MTCM research globally from 1951 to 2020. We used the volume of articles, authors, institutions, keywords, and so on as measurement points and discussed the development, trends, directions, and differences between Chinese and English literature. The findings obtained through this study will provide a more focused research perspective for subsequent scholars.

## 2. Data Acquisition and Analysis Methods

### 2.1. Data Sources and Processing Methods

ChP is not only he legal basis for the development, manufacture, operation, usage, and supervision of medicines but reflects the technology utilized for worldwide drug quality control [32]. Therefore, we chose 11 MTCM recorded in the 2020 version of the ChP as search subjects. All Chinese literature data for this study were obtained from the databases of the China National Knowledge Infrastructure (CNKI), Weipu (VIP), and WANFANG. English literature data were obtained from the Web of Science Core Collection (WoS). Search terms include “Marine materia medica,” “Marine traditional Chinese medicin,” “Shijueming, *Haliotids Concha*,” “Walengzi, *Arcae Concha*,” “Zhenzhumu, *Margaritifera Concha*,” “Geqiao, *Meretricis Concha Cyclinae Concha*,” “Haipiaoxiao, *Sepiae Endoconcha*,” “Haima, *Hippocampus*,” “Zhenzhu, *Margarita*,” “Hailong, *Syngnathus*,” “Haizao, *Sargassum*,” “Kunbu, *Laminariae Thallus Eckloniae Thallus*,” “Muli, *Ostreae Cconcha*.”

**Figure 1:**
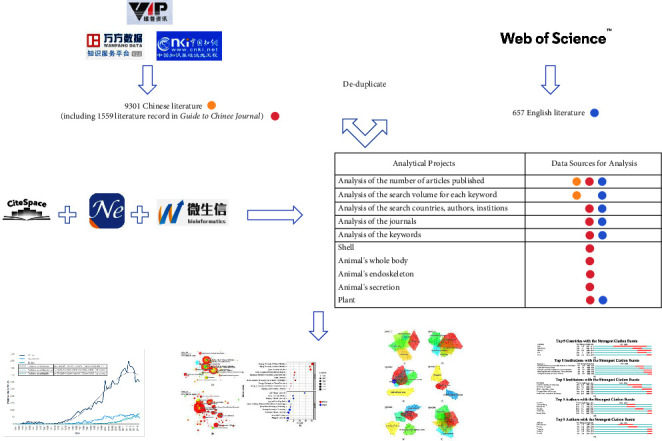
Flow chart of the analysis process.

The de-duplication procedure was accomplished by using NoteExpress (v3.5.0.9054) and CiteSpace 5.7.R2's “Data Import/Export-Remove Duplicate” function; bubble charts were plotted using https://www.bioinformatics.com.cn, a free online platform for data analysis and visualization.  Figure 1 explains the entire process of the study.

### 2.2. Research Methods and Related Parameters

Node type was set to “institution, country, or keyword” based on analytical projects; time slicing was set to 5 years; pruning function was set to “pruning sliced networks”; and other parameters remained the same by default.

The thickness of the different hues is related to the amount of publication, while the color of the nodes symbolizes the period. The “colder” color indicates an earlier publication date, while the “warmer” color indicates a later publication date, and the color of the lines between the nodes reflects the same meaning.

**Figure 2:**
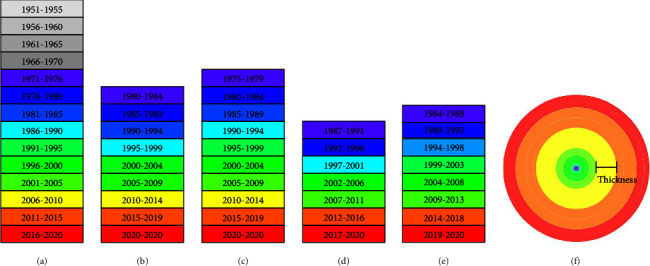
Description of node size and color. (a) Atlases of the Chinese literature, shells, animal's whole body, and plants (Chinese literature). (b) Atlases of the English literature. (c) Atlases of animals' endoskeletons (Chinese literature). (d) Atlases of animals' secretions (Chinese literature). (e) Atlases of plants (English literature). (f) Description of the annual rings.

In cooccurrence and cooperative atlases, the term “centrality” refers to intermediary centrality; if centrality >0.1, the node can be regarded as a critical node; “prominence value” judges the strength of the nodes present; in the cluster atlases, “cluster ID” is the cluster's serial number, the smaller the number, the larger the cluster sizes; “size” is the number of nodes in a cluster; “silhouette” (*S*) is the average profile value of the clusters, while *S* > 0.7, the cluster is considered to be well-clustered, all nodes are similar, and the results are convincing; the clustering module's “modularity” (*Q*) is its value; *Q* > 0.3 indicates that the clustering structure is important; in a bubble chart, the size of the bubble symbolizes centrality, while different colors stand for different data sources.  Figure 2 explains the meaning of the annual rings and the time represented by the color of the annual rings in different studies.

## 3. Results and Analyses

### 3.1. Analysis of Volume of Publication Published

Because there is so much retrieved Chinese literature, the quality is spotty, with numerous “folk recipes” and “informal prescriptions.” Except for analyzing the volume of literature published, other analytical project data were collected from the journal recorded in “Guide to Chinese Core Journals (GCCJ)” and the Chinese Journal of Marine Drugs.

#### 3.1.1. The Relationship between the Number of Publications and Particular Year

**Figure 3:**
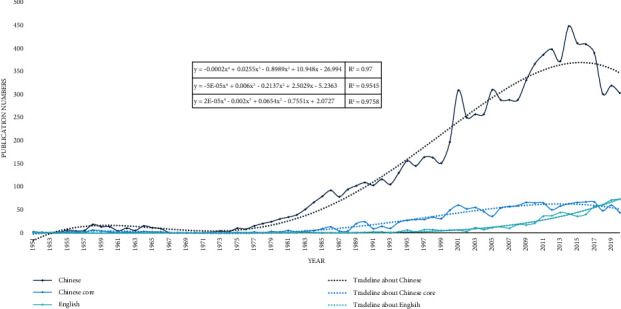
Annual publication numbers and trend lines of the English literature (cyan), Chinese literature (dark blue), and guide to Chinese core journals' literature (blue). The better the fit of the curve, the closer the *R*^2^ of the trend line equation is to 1; positioned at lines 150–162.

After de-duplication, a total of 9,958 publications were collected, including 9,301 Chinese publications (1,559 publications from GCCJ) and 657 English publications.  Figure 3 is the annual distribution trends of them. From the perspective of publication volume, the overall publication volume of Chinese literature is much higher than that of English literature, despite the fact that the number of publications in GCCJ is only marginally higher than that of English literature. From the perspective of the trend of publication volume, the publication volume of Chinese literature grew at a steady rate until the twenty-first century. From 2000 to 2015, the number of publications rose sharply, followed by a sharp fall, whereas the number of publications in the GCCJ has remained stable since the beginning of the twenty-first century, with no precipitous fluctuations. From 1980 to 2013, the volume of English literature progressively increased, remained steady in 2013, however, and increased dramatically in 2018, with the annual volume of literature surpassing that of the GCCJ.

As previously stated, the rapid development of the contemporary nautical and fishing industries has enabled people to better understand the sea. Due to the special growth environment of MTCM, the exploration of marine areas in China may have hit a snag since 2010. In comparison to Chinese literature, English literature is still in a phase of steady productivity, with more publications than GCCJ literature.

#### 3.1.2. Number of Publications per MTCM

**Figure 4:**
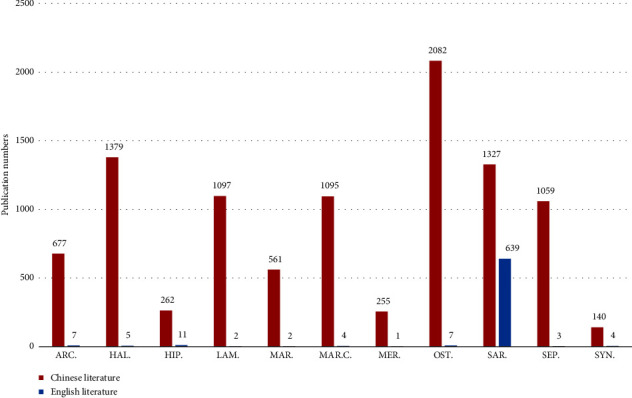
Statistics about the number of publications on 11 MTCM. SYN. = *Syngnathus*; SEP. = *Sepiae Endoconcha*; SAR. = *Sargassum*; OST. = *Ostreae Concha*; MER. = *Meretricis Concha Cyclinae Concha*; MAR.C. = *Margaritifera Concha*; MAR. = *Margarita*; LAM. = *Laminariae Thallus Eckloniae Thallus*; HIP. = *Hippocampus*; HAL. = *Haliotidis Concha*; ARC. = *Arcae Concha*; positioned at lines 174–188.

Information from Chinese and English literature was unified and summarized in NoteExpress software. We counted the amount of literature obtained for each search term separately and summarized it in WPS software before creating a double-column chart, and the results are shown in Figure 4. The search item “*Ostreae Concha*” had the largest number of publications in the Chinese literature, and it was also the most regularly prescribed in MTCM prescriptions [33]. The article “*From the usefulness and utilization of Ostreae Concha to the substitution of Ostreae Concha for keel bones*,” published by Pei in the Journal of traditional Chinese medicine served as the research starting point for *Ostreae Concha*. The major concentration of research in Chinese literature is on shell and botanical-based TCM; animal-based TCM such as “*Hippocampus*” and “*Syngnathus*” has received less attention because of a lack of resources and high prices. The chart's most striking result in English literature is that the result of the search term “*Sargassum*” (707) is higher than others. The experimental literature “*Antibiotic characteristics of Sargassum natans from Puerto Rico*,” published in the Journal of Pharmaceutical Sciences in 1961, was the starting point for the English literature investigation [34].

### 3.2. Cooperation Analysis of Research Countries, Authors, and Institutions

**Figure 5:**
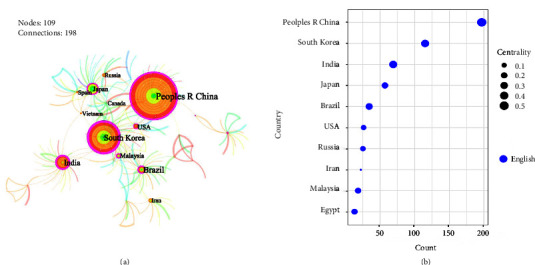
Countries' cooperation atlas and bubble chart based on the English literature. (a) Cooperative atlas of countries. (b) Bubble chart of the countries' characteristics based on the research data obtained from the English literature (top 10); positioned at lines 195–200.

To obtain a cooperation atlas and bubble chart, the network node was set to “country” (Figure 5); take note that the source of the data here is from WoS. The number of publications is the highest in China (197), followed by South Korea (115), India (69), and Japan (57), all of which have more than 50 publications. The centrality of China reached 0.55, indicating that China is an important location for information transfer in the field of MTCM research.

**Figure 6:**
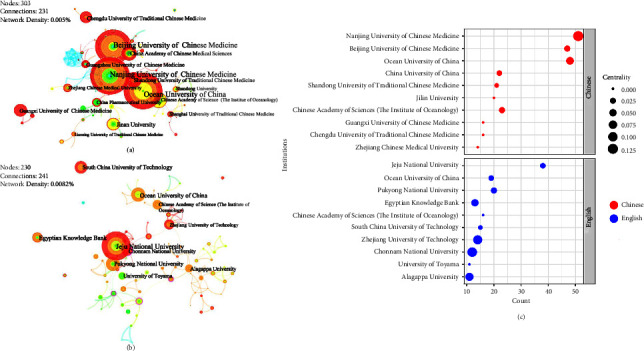
Institutional cooperation atlases and bubble charts based on the English and Chinese literature. (a) Cooperation atlas of institutions that have published the Chinese literature. (b) Cooperation atlas of institutions that have published the English literature. (c) Bubble chart of the institution characteristics for the research data obtained from the Chinese and English literature (top 10, respectively); positioned at lines 205–217.

We set the network node to “institution” and then filtered, merged, and renamed all institutions into first-class institutions and visualized the data results as shown in Figure 6. Nanjing University of Chinese Medicine, China (NJUCM) (51), and Jeju National University, South Korea (JEJUNU) (38), ranked first in the number of English and Chinese publications, respectively. Chinese institutions can be split into two categories: Chinese medical related universities and “marine” related universities. It is noteworthy that Ocean University of China, China (OUC), and the Chinese Academy of Sciences (The Institute of Oceanology), China (CASIO), have published numerous papers in both language journals. The centrality of NJUCM and Chonnam National University (CNU), South Korea, is all over 0.1, indicating that they are major communication nodes and have scientific authority in the field. In addition to these two institutions, the Egyptian Knowledge Banke (EKB) in Egypt, the University of Toyama in Japan, and Alagappa University in India were active in this field; these results matched the country analysis above. In Chinese literature, CASIO and OUC frequently appear, while other higher education institutions generally collaborate with their affiliates and clinical hospitals. Compared to institutions in Chinese literature, institutions in English literature cooperate more closely. Taken together, the MTCM area is centered on Chinese institutions led by NJUCM and South Korean institutions led by JEJUNU, all of which have professional teams and established research processes, as well as a wealth of expertise and excellent research skills.

**Figure 7:**
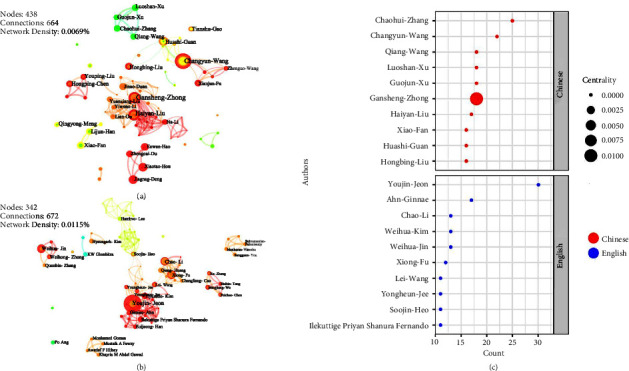
Author cooperation atlases and bubble charts based on the English and Chinese literature. (a) Cooperation atlas of authors who have published the Chinese literature. (b) Cooperation atlas of authors who have published the Chinese literature. (c) Bubble chart of the author characteristics for the research data obtained from the Chinese and English literature (top 10, respectively); positioned at lines 229–242.

As indicated in Figure 7, Chinese scholar Zhaohui-Zhang is the author with the most publications (25) in Chinese literature and routinely collaborates on *Hippocampus* and *Syngnathus* research with Zhang et al. [35–37]. Changyun-Wang (22) is a professor at OUC; he conducted some research studies on the characterization and microscopic identification of TCM [38] as well as the activity or structure of marine fungi [39, 40]. Youjin-Jeon, a South Korean scholar with the highest number of publications (30), is a professor at the College of Marine Biomedical Sciences, JEJUNU. He collaborated with Ginnae-Ahn and Fernando-Ilekuttige Priyan Shanura. [41, 42]. Chao-Li, a young professor at the South China University of Technology (SCUT), is the third person to publish the most English literature. He has worked with Xiong-Fu, Qiang-Huang, and Lianghuang-Cao on a number of occasions and conducted research studies on both sea cucumbers and *Sargussum* [43, 44]. Only Gansheng-Zhong's centrality reaches 0.1, as depicted in Figure 7(c), indicating that he was serving as a connector between authors.

To summarize, despite the fact that China is the source of MTCM with a significant number of publications, the collaboration network density of authors in English literature was higher than that of authors in Chinese literature. This finding is consistent with the previous findings on institutions.

### 3.3. Analysis of Journals

The top 10 lists of published journals in Chinese and English were obtained by using the “data analysis” function of NoteExpress (Tables 1 and 2). The most published Chinese journal is the Journal of Traditional Chinese Medicine (156), which was founded in 1955. The second place is the Chinese Journal of Marine Drugs (124), which is the first worldwide academic journal devoted to marine medicine; however, it has a somewhat lower compound impact in comparison. The Chinese journals studying MTCM, whose research areas focus on Chinese medicine, TCM, and pharmacology, are covered. The English journal with the highest number of publications is Marine Drugs (95), which was established at OUC and whose founding editor-in-chief is the professor Huashi-Guan. The overall quality of articles published in English journals is strong, and numerous high-level journals, such as Carbohydrate Polymer Technologies and Applications, are included in JCR Q1. The English literature consisted of approximately 200 periodicals focused on medicine, molecular biology, chemistry, fisheries, and other fields.

### 3.4. Analysis of Keywords

#### 3.4.1. Cooccurrence Analysis of Keywords

A keyword is a succinct expression of an article's fundamental substance, and to a certain extent, the high frequency of a term at a given time reflects the period's research hotspots [45]. Because different medicinal portions of the MTCM are utilized for different purposes and ways [46], we have therefore categorized the MTCM into 5 categories based on the sections of the medicine used [47] (according to 3.1.2, the keyword analysis of the English literature was limited to the category of plant because the search results of the English literature almost entirely circled around Sargassum):

- Category 1: Shell: *Haliotids Concha*, *Arcae Concha, Margaritifera Concha, Meretricis Concha Cyclinae Concha, Ostreae Concha*
- Category 2: Animal's whole body: *Hippocampus, Syngnathus*
- Category 3: Animals' endoskeleton: *Sepiae Endoconcha*
- Category 4: Animal's secretions: *Margarita*
- Category 5: Plant: *Sargassum, Laminariae Thallus Eckloniae Thallus*

**Figure 8:**
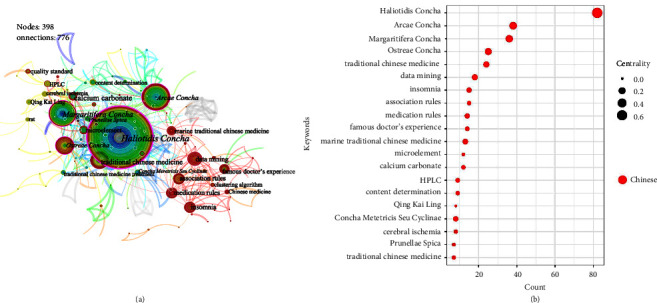
Keywords (category 1): cooccurrence atlas and bubble chart based on Chinese literature. (a) Cooccurrence atlas of keywords. (b) Bubble chart of the publication characteristics for the keywords about shell (top 20); positioned at lines 284–298.

Figure 8 depicts the difference in the number of publications between the result of 3.1.2 and this result: the previously counted ranking list shows *Ostreae Concha* at the top; however, this statistic shows *Haliotids Concha* at the top. As a result of this finding, we can deduce that an additional high-quality study on *Haliotidis Concha* has been undertaken. This TCM has a long history of use, and it was commonly pulverized and powdered to cure eye diseases [48]. Furthermore, it was often used to treat vertigo by Mr. Bohua-Kong, a famous old Chinese medicine practitioner [49]. Ma et al. [50] discovered that aqueous extracts of *Haliotidis Concha* can also decrease angiotensin-converting enzyme (ACE) activity and have a powerful and long-lasting antihypertensive impact. We can see that “data mining,” “determination of inorganic elements,” and “famous doctor's experience” are all prominent by observing the statistics on quantity and centrality in Figure 8(b). Due to the fact that calcium salts and microelements are the key chemical components of shellfish class MTCM, their contents are closely associated with the efficacy and quality of MTCM, and their contents are also closely tied to the origin, growing period, and marine biological environment [51, 52]. Beyond that, the combination of data mining and association rules is a novel technique to summarize and explore the medication rules of famous Chinese medicine practitioners.

**Figure 9:**
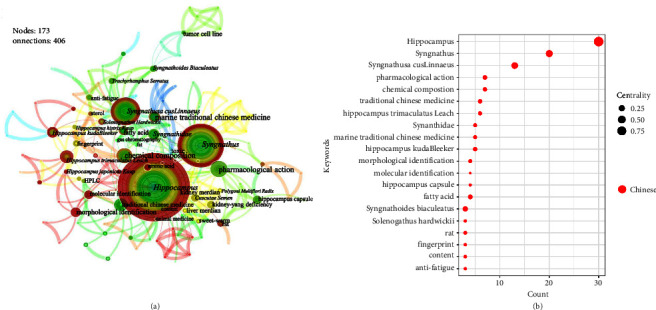
Keywords (category 2): cooccurrence atlas and bubble chart based on Chinese literature. (a) Cooccurrence atlas of keywords. (b) Bubble chart of the publication characteristics for the keywords about whole of animals (top 20); positioned at lines 305–316.

Figure 9 contains the keywords (category 2) cooccurrence atlas. Apart from the species nouns of *Hippocampus* and *Syngnathus*, the keyword “fatty acids” has a higher centrality than others. Both the *Hippocampus* and the *Syngnathus* contain 14 different types of fatty acids [53], particularly the unsaturated fatty acids ARA, EPA, and DHA, which are essential nutrients the human body cannot produce on its own [54, 55]. They play a significant role in maintaining body health and curing ailments, and their contents are frequently employed as a quality evaluation index [56]. In terms of pharmaceutical effects, “antifatigue” appears in the cooccurrence atlas, and research has verified that both the *Hippocampus* and the *Syngnathus* have antifatigue properties [57, 58]. Apart from these, “morphological identification” and “molecule identification,” both part of the identification process, indicate that scholars have conducted some discriminating research in this field.

**Figure 10:**
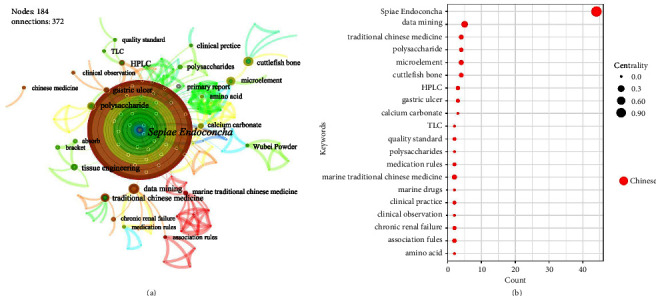
Keywords (category 3): cooccurrence atlas and bubble chart based on Chinese literature. (a) Cooccurrence atlas of keywords. (b) Bubble chart of the publication characteristics for the keywords about inner shell of animals (top 20); positioned at lines 321–327.

The keyword analysis result for category 3 is similar to that of category 1. Scholars often apply data mining methods and regard inorganic elements as research objects. Funnily enough, the keyword “polysaccharide” appears prominently in Figure 10. Although *Sepiae Endoconcha* is an animal bone, it also contains substances such as mucus, polysaccharides, and enzymes [59]. *Sepiae Endoconcha* polysaccharide (CPS-1) has been proven in studies to speed up the healing and repair of ulcer tissue as well as have a cytoprotective effect on the stomach mucosa [60, 61].

**Figure 11:**
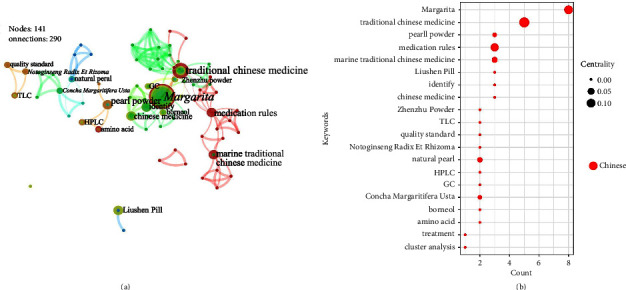
Keywords (category 4): cooccurrence atlas and bubble chart based on Chinese literature. (a) Cooccurrence atlas of keywords. (b) Bubble chart of the publication characteristics for the keywords about secretions of animals (top 20); positioned at lines 332–336.

*Margarita* is generated in the body by stimulation of bivalves as described in Chp [62], so we classify it in the animal secretion class MTCM. By observing Figure 11, scholars generally conducted research on Chinese patent medicines containing *Margarita* (e.g., Liushen Pill, Zhenzhu Powder), and it was often detected by chromatography (e.g., TLC, HPLC, or GC) [63, 64].

**Figure 12:**
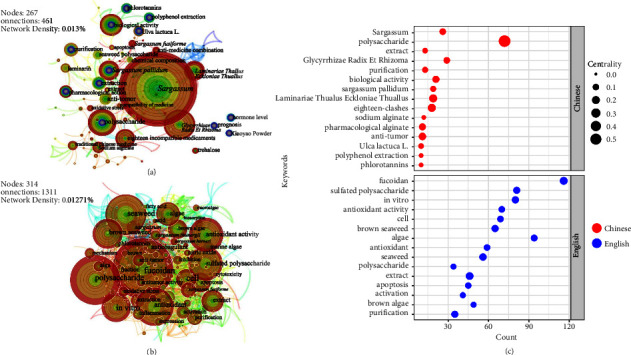
Keywords (category 5): cooccurrence atlas and bubble chart based on Chinese and English literature. (a) Cooccurrence atlas of keywords based on Chinese literature. (b) Cooperation atlas of keywords based on English literature. (c) Bubble chart of the keyword characteristics for the keywords for the research data obtained from Chinese and English literature (top 15 respectively); positioned at lines 341–357.

Figure 12 displays the keywords characteristics of category 5 in Chinese and English literature. The beginning of *Sargassum* research in China has emphases on its active ingredients (e.g., polysaccharides, sodium alginate [65]) and their effects while combined with other TCM (e.g., *Laminariae Thallus Eckloniae Thallus*, *Glycyrrhizae Radix Et Rhizoma* [66, 67]). Researchers have also looked into the extraction and pharmacological activity of polyphenolic compounds. In English literature, there is a substantial amount of research on fucoidan and seaweed polysaccharides. The high frequency keyword count of the passages showed that most of the English literature is based on cell research in vitro and rat experiments in vivo to verify its antitumor, anti-inflammatory, antioxidant, and other effects [68–70]. Figure 12(c) illustrated the disparities in the prominence between Chinese and English literature. The results of keyword analysis in Chinese literature, such as “*Sargassum*,” “*Laminariae Thuallus Eckloniae Thallus*,” and “eighteen-clashes,” show that the centrality of these words is over 0.1. The majority of English literature keywords have a centrality of 0.03∼0.08, with the centralities of “purification” and “algae” being greater than or equal to 0.1. This result indicated that the authors of the English literature have made a thorough study of *Sargassum* from every viewpoint, and the researchers have spent some time thinking about the extraction and purification processes.

#### 3.4.2. Cluster Analysis of Keywords

**Figure 13:**
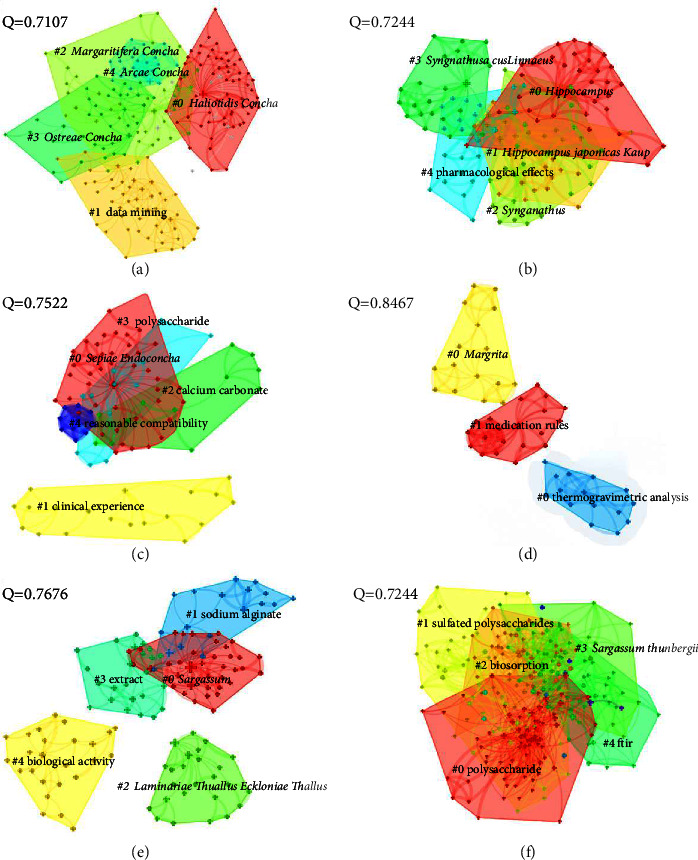
(a) Cluster analysis of shell class MTCM based on Chinese literature. (b) Cluster analysis of an animal's whole body class in MTCM based on Chinese literature. (c) Cluster analysis of the animal's endoskeleton class MTCM based on Chinese literature. (d) Cluster analysis of the animal's secretion class MTCM based on Chinese literature. (e, f) Cluster analysis of plant class MTCM based on Chinese and English literature.

Each category of keywords was administered to clustered analysis independently after being calculated by the LLR clustering algorithm, as illustrated in Figure 13, and the characteristics of each cluster are presented in Tables 3–8.

### 3.5. Analysis of Trends

#### 3.5.1. Burstiness Analysis of Countries, Authors, and Institutions

**Figure 14:**
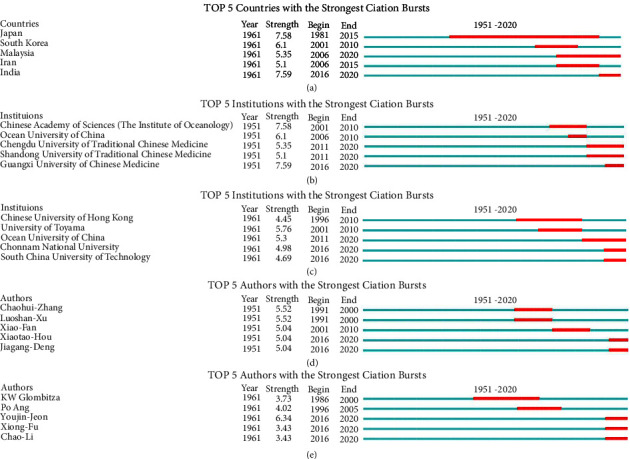
(a) Countries with the strongest citation bursts (top 5). (b) Institutions based on the Chinese literature with the strongest citation bursts (top 5). (c) Institutions based on the English literature with the strongest citation bursts (top 5). (d) Chinese authors with the strongest citation bursts (top 5). (e) English authors with the strongest citation bursts (top 5); positioned at lines 386–406.

Five burstiness atlases were collected after applying the burstiness function (Figure 14). In Figure 14(a), Japan has the highest prominence value (14.17), followed by South Korea (6.25), but Malaysia and India were still in the emergence time range until 2020, which indicated that they were experiencing a research boom. Figures 14(b) and 14(c) display the statistics for institutions. The prominence value of Guangxi University of Chinese Medicine (GXTCMU) (7.59) is higher; this institution has seen a boom in publication since 2016 and keeping highly product. OUC is listed in both statistics atlases, and Chengdu University of Traditional Chinese Medicine (5.35), Shandong University of Traditional Chinese Medicine (5.1), CUN (4.98) and SCUT (4.69) also have high prominence values and have conducted research in this field recently. In the burstiness atlases of authors (Figures 14(d) and 14(e)), Zhaohui-Zhang (5.52), and Luoshan-Xu (5.52) have the highest prominence value. They are not affiliated with the same institution but collaborate closely to do research on *Hippocampus* and *Syngnathus*. Chen et al. also have a high prominence value (5.04), they are in the same research team with Erwei-Hao at GXTCMU. This conclusion substantially confirmed the findings in Figure 14(b), and research progress and safety evaluation of MTCM are part of their research [71]. You-jin Jeon is an author in English literature with the highest number of publications and the largest prominence value (6.34). Xiong-Fu (3.43) and Chao-Li (3.43), who work at SCUT, although both of them do not have high prominence value, there is no denying that their contributions have enhanced the prominence value of their institution, and they have been active in recent years.

#### 3.5.2. Leapfrog Analyses of Keywords

**Figure 15:**
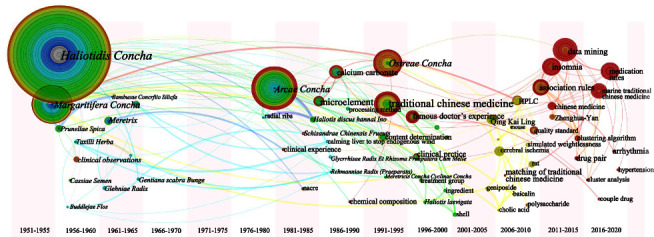
Keyword leapfrog atlas about category 1 in Chinese literature; positioned at lines 415–419.

**Figure 16:**
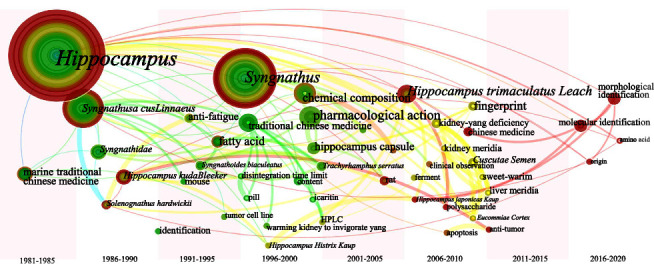
Keyword leapfrog atlas about category 2 in Chinese literature; positioned at lines 422–428.

**Figure 17:**
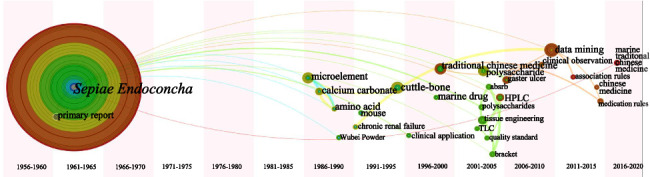
Keyword leapfrog atlas about category 3 in Chinese literature; positioned at lines 431–435.

**Figure 18:**
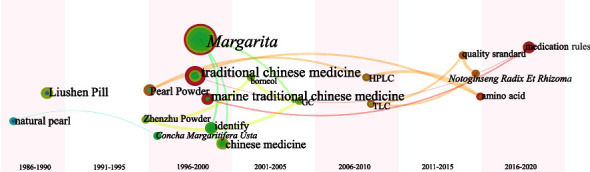
Keyword leapfrog atlas about category 4 in Chinese literature; positioned at lines 438–444.

**Figure 19:**
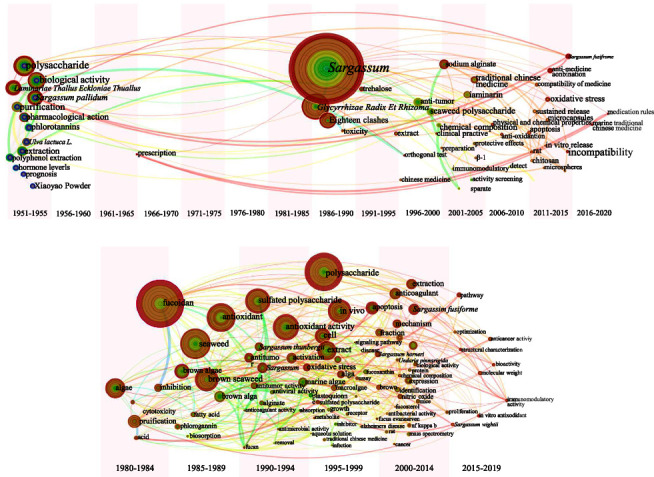
(a) Keyword leapfrog atlas about category 5 in Chinese literature. (b) Keyword leapfrog atlas about category 5 in English literature; positioned at lines 447–458.

Keyword leapfrog atlases were obtained by setting the presentation to “Timezone View” (Figures 15–19).

Figure 15 shows that *Haliotidis Concha* and *Margaritifera Concha* appeared earlier. MTCM research on shellfish class was limited to a single TCM from 1950 to 1980, and researchers began to explore ingredients in 1980. The emergence of the keywords “data mining” and “association rules” around 2010 suggests that the application of various algorithms to explore medication rules became common.

The starting point of the node leap is presented in Figure 16. After 1980, scholars conducted some research on the antifatigue effect of *Hippocampus* and *Syngnathus* around 1990, then they began to study the therapeutic impact of “warming the kidney to invigorate yang” until 1995. Fatty acids have been studied for longer and in greater depth, whereas amino acids have only recently been investigated. After 2015, due to the paucity of *Hippocampus*, research on morphological identification, molecular identification and origin has sprung up.

By observing the leapfrog atlas (Figure 17), the research on animal's endoskeleton class (*Sepiae Endoconcha*) MTCM was also started earlier, and the research on it was relatively single until 1980. Microelements, calcium carbonate, and amino acids have been researched since 1980, while polysaccharide components have been researched since 2000. Similarly, around 2010, research on data mining emerged.

The leapfrog atlas of animals' secretion class MTCM (Figure 18) displays fewer keywords; the result is organized into three periods depending on research feature. Early stage (before 2000): pharmacological effects or clinical observation studies on *Margarita*-containing TCM compounds. Mid-term (2000–2010): application of chromatography technologies for chemical composition detection or quality control. Later stage (after 2010): analysis of pharmaceutical rules and determination of amino acid content in Margarita using various algorithms.

As illustrated in Figure 19, there are some variations between the Chinese and English literature on the research points and timing of plant class MTCM. A hiatus in Chinese literature research on plant class MTCM existed from 1950–1990, and along with the increased attention to *Sargassum* worldwide, Chinese researchers made a qualitative and quantitative jump in their research on *Sargassum* starting in 1990. Prior to 2000, the research in China was limited to the efficacy of crude extracts, and after 2000, studies on the mechanism of action were carried out successively. Earlier studies in English literature had deeply studied polysaccharides and fucoidan for extended periods of time, revealing antitumor, antioxidant, anti-inflammation, and other effects. Until 2010, its modulatory function on the immune system and anticoagulant effects were also excavated. Since 2015, scholars have started to investigate at the molecular structural characterization level and spare no effort to optimize the extraction procedures.

## 4. Discussion

This study applied CiteSpace 5.7.R2 software from the perspectives of publication number, author, institution, and keyword to research and summarize the publication situation, country-author-institution cooperation, research path evolution, and key research directions of China and other countries' research in the field of MTCM.

Through the analysis of the cooperation between countries, it is obvious that China was widely recognized by international peer researchers for producing high-quality and co-citated literature. South Korea, India, and Japan are all coastal countries with favorable geographic conditions and abundant natural resources. Especially in South Korea, where the aquatic industry is well developed, this condition provides a strong technological foundation for the burgeoning businesses of marine medicine and healthy food [72].

The cooccurrence analyses of institutions and authors showed that institutions in China and South Korea outperformed those in other countries, but the lower density of collaboration in China is a lateral expression of a lack of communication between institutions. This can be classified for two reasons: first, Chinese academics tend to conduct research from a theoretical standpoint, yet relevant records are scarce and vague, which makes research more challenging. Second, there is a big fraction of *Sargassum*-related topics in English literature, which is the most important reason for the high density of collaboration. To balance this situation, the Chinese government, research institutions, and scholars could consider the following perspectives.

We have three recommendations for the related departments. First of all, now that only 11 MTCM are recorded in the ChP, associated departments should strengthen the resource census and thoroughly investigate the resource base, thereby boosting more research-worthy MTCM incorporated into the ChP, such as sea cucumbers, sea urchins, and sea stars, which have established mature research systems is necessary. Secondly, through keyword cooccurrence analyses, we found the resources of some species are being depleted; for example, *Hippocampus* is currently in serious decline and has suffered depletion of its natural resources; it is now listed on the IUCN Red List and the China species Red List [73]. Hence, it is imperative to avoid the circulation of pseudo-mixed products in the market through morphological identification, molecular identification, and content determination. Scarce resources, such as *Hippocampus* and *Syngnathus*, should be cultured in captivity or introduced as new species, prevention of species mixing, stopping counterfeit and substandard medicines to affect the effectiveness and safety of clinical use of MTCM. Finally, establishing quality standards and clinical application guidelines through qualitative and quantitative analysis of a component of MTCM, which is common in our summarized study. We found that the quality standard of MTCM is uneven, small amount of them have an easy physical or chemical identification standard and few of them have a content determination, even five different shellfish MTCM but using the same calcium carbonate content determination standard [52]. Therefore, related departments should designate a development strategy for MTCM and establish a standardized technical system.

As for the advice to institutions and scholars, the following perspectives can be referred to. One of them is: Chinese scholars make up the majority of the field, and they are particularly interested in following up on information recorded in ancient books, which is noteworthy because there is variability between the literature and ancient books about MTCM [74]. This requires the scholars to apply modern information technology to delve deeply into the literature and historical materials; “data mining” and “affiliate rules” present in our study fit with this phenomenon. Now, only 7 MTCM have relatively complete herbal texts [35, 74–78], necessitating a thorough examination of other MTCM foundation sources. Another one is: “Theory of medicinal nature” frequently appears in our cooccurrence atlases; Chinese medicinal nature is the intrinsic characteristics that distinguish TCM from other medicines [74]. We can consider that it is simpler to target key points and improve research efficiency when traditional oriental medical theory and modern western pharmaceutical theory collide.

Based on the cooccurrence, clustering, and leapfrog analyses of keywords in Chinese literature, we found MTCM has specific emphases in China, such as process methods, the medicinal nature of TCM, textual or visual identification of pseudo-mixes, etc. The processing of TCM, as a unique traditional pharmaceutical technique in China, is an essential tool and an important part of ensuring the clinical efficacy of Chinese medicine [79, 80]. When MTCM is processed, its medicinal effects, contents of some components, and medicinal properties may change [81–83]. We also concluded that scholars usually research MTCM by applying analytical tools, digging into the medication rules, and summarizing the experiences of famous doctors. After 2010, the “affiliation rules” cluster emerged, which coincides with the previous statement. Affiliation rule is a technique for mining valid relationships from huge amounts of valuable data. “Data mining” is an emerging discipline that combines theories and techniques from fields such as mathematics, statistics, and artificial intelligence. The combination of these two has already been widely used in the TCM field [84, 85]. By classifying and analyzing the MTCM of different medicinal portions, we can see that mineral-based MTCM (shell, an animal's endoskeleton, and an animal's secretion) and inorganic elements and microelements were the focus of research; plant-based MTCM research in China concentrate on thyroid disorder or gall goiter, while research in other countries focuses on cancer or inflammation, which is another reminder of the differences between modern and traditional medicine and the need for integration. Animal-based MTCM employs the entire body of the animal as the source of medicine, and both have the pharmacological effect of “warming the kidney to invigorate yang” from a TCM perspective, while from a western medical point of view, they have an antifatigue effect.

Being different from the hotspots of research in Chinese literature, English literature focuses on *Sargassum* as a whole, preferring to explore the action mechanisms, extraction, and purification of active substances, most well studied for antioxidant and antitumor properties, but it is not limited to these two applications absolutely. Based on keywords analyses, we found that researchers often take the regulation of the signaling pathway by medicine as the starting point. Take the classical NF-*κ*B signaling pathway as an example, inflammatory stimuli activate the NF-*κ*B inducible transcription factor, which is found ordinarily in the cytoplasm as an inactive trimer and translocated subsequently from the cytoplasm to the nucleus, increasing the development of inflammation or cancer [86–88]. Yoon et al. [89] found that Sargachromanol G, which is isolated from the brown alga *Sargassum siliquastrum*, can inhibit the conversion of RAW 264.7 to osteoblasts by activating the NF-*κ*B signaling pathway. By observing the keyword leapfrog and clustering analysis atlases, we have found that the technical combined application of chromatography, mass spectrometry, and spectroscopy is becoming increasingly common [90, 91]. Combining the ability of chromatography to separate complex samples with the high sensitivity of mass spectrometry or spectroscopy to determine relative molecular mass and structural information, these facilitate the investigation of the active ingredients and structures in MTCM in more depth and optimize the extraction process [92, 93].

Taken together with the discussion of keywords, it is explicit that the study of MTCM in Chinese literature started earlier while the study in English literature started later, but the pharmacodynamic mechanism is clearly defined. Currently, the main idea underlying MTCM applications in both Chinese and English literature is to screen medication lead compounds from marine resources, then developing and changing them structurally. Lastly, turn them into approapriate therapeutically medicines.

Based on the results of the analysis, a particularly large number of scholars have been studying *Sargassum* in recent years, in light of this, there are three proposals for subsequent scholars: first: “sulfated polysaccharide” has a prominent place in keyword analyses and it is also verified that appropriate sulphation changes have been proven to improve the biological activity of seaweed polysaccharides [94]. It has also been verified that selenization leads to some changes in the monosaccharide composition, molecular weight, and surface morphology of seaweed polysaccharides, which exhibit more pronounced inhibition of *α*-glucosidase activity in a noncompetitive inhibition type [95]. Continuing on from this point, scientists can consider more novel ways to modify *Sargassum* polysaccharides, which may cause unanticipated profits. Second: the optimization of extraction process is a hot research topic in recent years; however it is well known that the extraction of seaweed polysaccharides is not high and the purification process needs to be optimized [96]. So, the other components of the *Sargassum* species, such as inorganic elements and polyphenols, are likely to play a positive role. Third: MTCM is originated in China, although Chinese scholars and institutions are particularly engaged in this sector, the density of cooperation between institutions and authors is not excellent. As a result, cutting-edge institutions including NJUCM, CASIO, OUC, SCUT, etc. should make breakthroughs from the existing research base, strengthen communication and coordination, and collaborate closely to achieve more valuable and credible achievements.

Under the network mapping of the keyword analysis, we found the appearance of many disease symptoms reflected the unique activities and potential of MTCM. More than half of the MTCM have pharmacological activities such as antioxidation, antitumor and antiviral, according to researches [97]. “Antioxidation” or “oxidative stress” can be seen everywhere in the keyword analysis atlases, which show that MTCM has a most outstanding antioxidant effect. Increased oxidative stress alters lipids, DNA, and proteins, leading to cellular inflammation and programmed cell death, which play an irreplaceable role in physiopathological conditions [98]. *Sargassum ilicifolium* crude lipid extracts demonstrated antioxidant activity in an in vitro experiment [99]. Macroalgae are also considered to be an important source of secondary metabolites and macromolecules with antioxidant activity [100]. Cancer has become a global public health problem over the last few decades [101], and MTCM has made many inspiring achievements and formed a therapeutic system in the field of cancer treatment [102]. The extracts of *Syngnathus* have been found to inhibit tumor cell proliferation, reduce the tumor formation rate, and prolong the survival time of mice [103, 104]. Fucoidan from *Saccharina japonica* and *Undaria pinnatifida* have a significant effect on antitumor activity as well [105]. Undeniably, due to the multitargeted nature of MTCM (especially compound preparations), it has been proven to be effective in treating a wide range of viral diseases [106]. Numerous components in *Sargassum,* including ACE inhibitory peptides and soluble dietary fibers (e.g., fucoidan, porphyran, etc.), could minimize the ACE dominance caused by SARS-CoV-2 infection, enhancing the effectiveness of the COVID-19 vaccine [107]. TCM compound preparations, which contain MTCM, have been verified. For example, *Margaritifera Concha* has now been demonstrated to be effective against influenza and herpes viruses [108]. Pharmacological effects, such as inflammation [109], an anticoagulant effect [110], an antiallergic [111], an antibacterial [112], and even the treatment of obsessive-compulsive disorder [113], etc., have all been verified.

## 5. Conclusion and Expectation

As a type of TCM with a unique growth environment, an increasing number of experts are conducting studies on MTCM, and the number of publications is growing annually. China, as a pioneer in this field, has carried out a multifaceted, long-term study based on traditional theories (e.g., the processing theory of TCM and the theory of medicinal nature). Research points in other countries are less extensive but more in-depth (e.g., action mechanisms, extraction, and purification of active substances) than research points in China. Meanwhile, the modernization of Chinese research has been aided by scientific thoughts from other countries. By separating the analysis of MTCM according to different medicinal parts, we also found differences in research hotspots between them. Interdisciplinary research can help maximize the benefits of MTCM in the battle against diseases such as cancer, oxidative stress, inflammation, and viral infection. According to recent research hotspots and trends, the return of ancient TCM books, the updating of analytical techniques and tools, the organic integration of the “holistic concept” of traditional medicine and the “individual concept” of modern medicine, the exploration and modification of active ingredients, and the in-depth study of marine organisms (e.g., algae) are all top priorities for all scholars.

The vast majority of MTCM uses plants and animals grown in the ocean as a source of medicine. MTCM as a medicine source is the main difficulty and obstacle to the development of marine medicine, but it can also be the greatest advantage and the strongest foundation for marine medicine [114]. At this stage, accelerating the development and industrialization of marine Chinese medicine is a major problem. From the emergence of Chinese first marine drug (Poly Saccharide Sulphate, PSS®), which was extracted from natural *Sargassum* in 1985 [115], to the proposal of the “Blue Drug Store” development plan in 2016, all these inspiring results showed that MTCM, which originated in China, is merged with modern concepts and embodies a wealth of values. It is undoubtable that “blue pharmaceutical” will become a leading high-tech industry as a result of further in-depth research and development in the field of marine medicine.

## Figures and Tables

**Table 1:** Top 10 published journals based on the CNKI, VIP, and WANFANG.

Rank	Journal name	Volume of publication	Compound IF (2021)
1	Journal of Traditional Chinese Medicine	156	2.313
2	Chinese Journal of Marine Drugs	124	0.669
3	Lishizhen Medicine and Materia Medica Research	115	1.136
4	China Journal of Chinese Materia Medica	108	3.076
5	Journal of Chinese Medicinal Materials	101	1.309
6	Chinese Traditional Patent Medicine	98	1.668
7	Chinese Traditional and Herbal Drugs	89	3.16
8	Chinese Journal of Experimental Traditional Medical Formulae	61	3.038
9	China Journal of Traditional Chinese Medicine and Pharmacy	41	2.083
10	Journal of Basic Chinese Medicine	35	1.204

**Table 2:** Top 10 published journals based on the WoS.

Rank	Journal name	Volume of publication	IF (2021–2022)
1	Marine Drugs	95	6.085
2	International Journal of Biological Macromolecules	84	8.025
3	Carbohydrate Polymer Technologies and Applications	46	10.723
4	Food Chemistry	21	4.004
5	Journal of Agricultural and Food Chemistry	21	9.31
6	Phytochemistry	20	5.895
7	Molecules	17	4.927
8	Food & Function	16	6.317
9	Natural Product Research	14	2.488
10	Chemistry of Natural Compounds	13	0.830

**Table 3:** Cluster information about shell class MTCM based on Chinese literature.

Cluster ID	Cluster name	Size	Silhouette	Mean (year)	Top terms (LLR, *p* value)
0	*Haliotidis Concha*	59	0.942	1984	*Haliotidis Concha*; *Arcae Concha*, etc.
1	Data mining	49	0.964	2014	Data mining; affiliation rules, etc.
2	*Margaritifera Concha*	40	0.889	1989	*Margaritifera Concha*; HPLC, etc.
3	*Ostreae Concha*	36	0.891	2003	*Ostreae Concha*, calcium carbonate, etc.
4	*Arcae Concha*	28	0.974	1988	*Arcae Concha*; *Meretricis Concha Cyclinae Concha*, etc.

**Table 4:** Cluster information about animal's whole body class MTCM based on Chinese literature.

Cluster ID	Cluster name	Size	Silhouette	Mean (year)	Top terms (LLR, *p* value)
0	*Hippocampus*	24	0.918	2008	*Hippocampus*; kidney yang deficiency, etc.
1	*Hippocampus japonicas Kaup*	23	0.83	2008	*Hippocampus kudaBleeker*; morphological identification, etc.
2	*Syngnathus*	20	0.892	2005	*Syngnathus*; clinical observation etc.
3	*Syngnathusa cusLinnaeus*	20	0.918	1997	*Syngnathusa cusLinnaeus*; fatty acid, etc.
4	Pharmacological effect	18	0.826	2002	Chemical composition, hippocampus capsule, etc.

**Table 5:** Cluster information about animal's endoskeleton class MTCM based on Chinese literature.

Cluster ID	Cluster name	Size	Silhouette	Mean (year)	Top terms (LLR, *p* value)
0	*Sepiae Endoconcha*	43	0.99	2004	*Sepiae Endoconcha*; adsorption, etc.
1	Clinical experience	19	0.999	2010	Clinical experience; logistic regression, etc.
2	Calcium carbonate	13	0.978	2002	Chinese medicine treatment; biominerals, etc.
3	Polysaccharide	12	0.965	2010	Polysaccharide; biological activity, etc.
4	Reasonable compatibility	10	0.965	2010	Reasonable compatibility; endomycin, etc.

**Table 6:** Cluster information about animal's secretion class MTCM based on Chinese literature.

Cluster ID	Cluster name	Size	Silhouette	Mean (year)	Top terms (LLR, *p* value)
0	Margarita	20	0.998	2001	*Margarita*; toxicology, etc.
1	Medication rule	16	0.96	2017	Medication rule; visual fatigue, etc.
2	Thermogravimetric analysis	15	0.961	2002	X-ray diffraction; quality analysis, etc.

**Table 7:** Cluster information about plant class MTCM based on Chinese literature.

Cluster ID	Cluster name	Size	Silhouette	Mean (year)	Top terms (LLR, *p* value)
0	*Sargassum*	38	0.938	2003	*Glycyrrhizae Radix et Rhizoma*; *Sargassum*, etc.
1	Sodium alginate	25	0.844	2007	Sodium alginate; microcapsules, etc.
2	*Laminariae Thallus Eckloniae Thallus*	25	0.985	2004	*Laminariae Thallus Eckloniae Thallus*; laminarin, etc.
3	Extraction	24	0.857	1995	Extraction; purification, etc.
4	Biological activity	18	0.955	1993	Biological activity; phlorotannins, etc.

**Table 8:** Cluster information about plant class MTCM based on English literature.

Cluster ID	Cluster name	Size	Silhouette	Mean (year)	Top terms (LLR, *p* value)
0	Polysaccharide	73	0.743	207	Polysaccharide; structure, etc.
1	Sulfated polysaccharide	47	0.665	2012	Sulfated polysaccharide; anti-inflammatory, etc.
2	Biosorption	44	0.832	2005	Biosorption; alginate, etc.
3	*Sargassum thunbergii*	39	0.813	2006	*Sargassum thunbergii*; *Endophytic fungus*, etc.
4	Ftir	35	0.832	2007	Ftir; Fick's second law, etc.

## Data Availability

Data sharing is not applicable to this research. All data resources were derived from domain resources: https://www.cnki.net/; https://www.wanfangdata.com.cn/index.html; https://lib.cqvip.com/ and https://www.webofscience.com/wos/woscc/advanced-search.
